# Progress of heparanase in septic cardiomyopathy: A review

**DOI:** 10.1097/MD.0000000000038901

**Published:** 2024-08-16

**Authors:** Di Chen, Lin-Jun Wang, Hong-Lei Li, Fei Feng, Jian-Chun Li, Liping Liu

**Affiliations:** a The First Clinical Medical School of Lanzhou University, Lanzhou, Gansu, P. R. China; b Departments of Emergency Critical Care Medicine, The First Hospital of Lanzhou University, Lanzhou, Gansu, P. R. China.

**Keywords:** fibrosis, heparanase, microcirculatory dysfunction, mitochondrial dysfunction, septic cardiomyopathy

## Abstract

Septic cardiomyopathy (SCM) is a severe complication caused by sepsis, resulting in a high mortality rate. The current understanding of the pathogenic mechanism of SCM primarily involves endocardial injury, microcirculation disturbance, mitochondrial dysfunction and fibrosis. Heparanase (HPA), an endo-β-D-glucuronidase, has been implicated in inflammation, immune response, coagulation promotion, microcirculation disturbance, mitochondrial dysfunction and fibrosis. Therefore, it was hypothesized that HPA may play an important role in the pathogenesis of SCM. The present study provides a summary of various pathophysiological changes and mechanisms behind the involvement of HPA in SCM. It also presents a novel perspective on the pathogenic mechanism, diagnosis and treatment of SCM.

## 1. Introduction

Sepsis is a clinical syndrome characterized by an imbalanced response of the body to an infection, leading to organ dysfunction. Its mortality rate is notably high (≤30%).^[1]^ Within sepsis cases, the occurrence of septic cardiomyopathy (SCM) ranges from 13.8% to 40.0%,^[2]^ with mortality rates of 70% to 90%.^[3]^ A previous clinical trial revealed that risk factors for SCM included a history of diabetes or heart failure, younger age, higher levels of NT-pro B-type natriuretic peptide (NT-proBNP), positive blood culture and lower left ventricular ejection fraction (LVEF).^[4]^ However, there are currently no clear criteria for the diagnosis of SCM. In 2018, Beesley et al^[5]^ defined SCM as a decrease in LVEF and ventricular dilatation during sepsis. However, LVEF is gradually being recognized as an indeterminate index of cardiac function due to its dependency on load conditions. Some researchers have described SCM as a syndrome of cardiac dysfunction that is not related to ischemia, and includes left and right ventricular (RV) dysfunction, reduced ventricular contractility and left ventricular dilatation.^[5–7]^ Early diagnosis of SCM mainly relies on LVEF monitoring of ventricular systolic function, but ~1/5 of patients with SCM may also develop diastolic dysfunction.^[4]^ It has been reported that 20% to 60% of patients with SCM first experience temporary and reversible reduction in diastolic function through echocardiography, which is accompanied by systolic dysfunction and ventricular dilation.^[8]^ The time required to recover diastolic function is much longer than that of systolic function.^[9]^ It has been shown that speckle tracking echocardiography is more effective in diagnosing SCM,^[10]^ and, among the various strain measurement indices, global longitudinal strain (GLS) is the most commonly used.^[11]^

GLS is influenced by the preload and afterload of the heart, but it is less affected compared with LVEF. Therefore, GLS is often recommended as a new method for evaluating left ventricular function and detecting impaired cardiac function at an earlier stage. However, its clinical application is still limited.^[12]^ Recently, there has been an increasing focus on the diagnosis and prognosis of SCM in relation to RV dysfunction.^[13]^ Since different studies have used different techniques for assessing RV dysfunction, including multiparameter approaches, such as echocardiographic measurements and RV cardiac output, this difference in definition affects the incidence of RV dysfunction in patients with sepsis.^[14]^ Since the measurement indexes of echocardiography are greatly affected by subjectivity, it hinders the diagnosis of SCM. Primary cardiomyocyte injury markers, such as BNP and cardiac troponin I are commonly used as diagnostic reference indicators for SCM^[15]^; however, their sensitivity and specificity are relatively weak, making them inadequate for early intervention in SCM.^[16]^ Previous studies demonstrated that a combination of heart-type fatty acid binding protein, pregnancy-associated plasma protein-A and myeloperoxidase can serve as a reliable indicator for monitoring myocardial injury and predicting prognosis in patients with SCM.^[17,18]^ However, despite its potential, this biomarker combination is not yet widely utilized in clinical practice. Consequently, there remains a significant dearth of specific biomarkers for diagnosing SCM, and therefore it is of utmost importance to identify more sensitive and specific markers for SCM.

**Figure 1.**
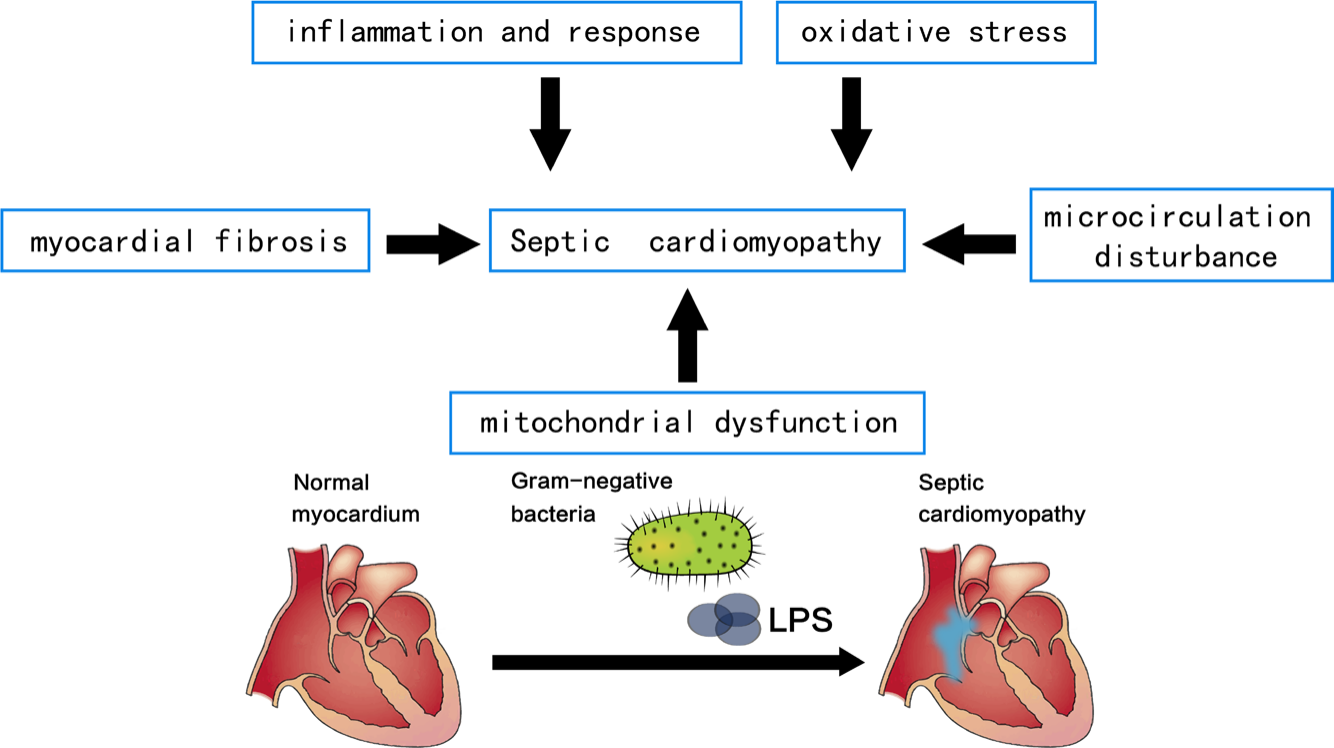
Selected from carbone F et al septic cardiomyopathy: From pathophysiology to the clinical setting. Cells. 2022 Sep 11;11(18):2833.

The pathogenic factors of SCM are complex and involve several factors, including inflammation-induced endocardial damage, disturbances in microcirculation, mitochondrial damage and excessive activation of sympathetic nerves (Fig. 1). Signals released by damaged host tissues, known as damage-associated molecular patterns, and pathogens, known as pathogen-associated molecular patterns, along with toll-like receptors (TLRs), activate various cellular pathways such as nuclear factor-κB and mitogen-activated protein kinase (MAPK).^[19,20]^ This activation leads to the release of pro-inflammatory cytokines and the destruction of myocardial endothelial cells. Lipopolysaccharide (LPS) and downstream inflammatory factors, such as interleukin-1β (IL-1β), tumor necrosis factor-α (TNF-α), complement 5a with anaphylactic toxins, and reactive oxygen species (ROS), can disrupt intracellular currents. This disruption can result in disturbances in calcium balance, leading to systolic or diastolic disorders.^[19,21]^ The activation of myocardial endothelial cells, stimulated by infection, leads to an increased release of inflammatory factors, which results in the overexpression of cell adhesion molecules and the shedding of endothelial glycocalyx (eGC), which impairs endothelial barrier function. Consequently, interstitial edema and myocardial cell apoptosis occur, along with the production of a hypercoagulable state and the induction of microvascular circulatory disorders.^[22]^ Mitochondria are crucial for maintaining normal cardiac function.^[22]^ In particular, lipid metabolism plays a significant role in mitochondrial function.^[23]^ During sepsis, certain fatty acid oxidases are inhibited, causing disruption in lipid metabolism and resulting in mitochondrial dysfunction,^[24–26]^ which leads to various morphological and functional alterations, such as interruption of oxidative phosphorylation, impaired mitochondrial respiration rate, generation of mitochondrial free radicals, reduction in mitochondrial membrane potential, inadequate ATP production, compromised autophagy and apoptosis. These changes significantly impact myocardial function.^[6]^ Downregulation of myocardial adrenergic receptor expression during sepsis has gained attention.^[27]^ Prolonged sympathetic activation can hinder cardiac contractility by causing adrenergic G protein coupling to switch to an inhibitory response. Additionally, decreased sympathetic stimulation may negatively affect cardiac filling time.^[28]^ In addition, fibrosis significantly contributes to the development of SCM.^[29]^ Cardiac fibroblasts release various pro-fibrotic factors through paracrine function, which accelerates the remodeling of the extracellular matrix (ECM). This further promotes fibrosis, resulting in ventricular dilation and myocardial injury.^[30,31]^ Therefore, the pathogenesis of SCM is primarily associated with endocardial inflammatory response, microcirculation disturbance, mitochondrial dysfunction and fibrosis. In the management of SCM, anti-infective therapy, fluid resuscitation, and organ support therapy are key treatment modalities. Antibiotic selection should aim for broad coverage of bacterial flora, including drug-resistant strains, fungi, and viruses. Higher antibiotic doses may be necessary to reach target drug concentrations.^[32]^ Early studies focusing on inflammatory factor-targeted treatments for sepsis, such as interleukin-1 receptor antagonists and tumor necrosis factor antibodies, have demonstrated significant therapeutic benefits in clinical settings.^[33]^ The HDL mimetic CER-001 has shown promise in alleviating various sepsis symptoms and potentially protecting organ function. Studies indicate that treatment with CER-001 can lower inflammatory factor levels, modulate immune responses, and enhance patient survival rates in sepsis cases.^[34]^ Fluid resuscitation is essential in the treatment of sepsis as it enhances tissue perfusion and oxygen supply, increases blood return to the heart, and boosts effective circulating blood volume.^[33]^ In recent years, the application of extracorporeal blood purification technology in patients with sepsis complicated by multiple organ dysfunction has been extensive, especially in patients with acute kidney injury, which is more beneficial for the hemodynamic stability and fluid balance of septic patients.^[35]^ Researchers have proposed that the main target of blood purification is to remove inflammatory factors, pathogens, endotoxins, and other damage-related pattern molecules in the bloodstream. However, due to the lack of unified guidance methods, more clinical randomized controlled trials are needed to prove the timing of initiation or cessation of different devices, treatment methods, etc, in order to improve the survival rate of septic patients.^[36]^

Heparanase (HPA), the only endogenous β-D-glucuronidase in mammals, is recognized as a significant controller of inflammatory diseases.^[37]^ HPA releases a diverse array of cytokines, enzymes, and lipoproteins by cleaving the HS chain of heparan sulphate proteoglycan (HSPG). Furthermore, HPA modulates its functions via histones, which are present both intra- and extracellularly and have the ability to release proteins with positive charges. HSPG, being one of the most negatively charged biopolymers, along with histones, can interact with GAG chains through charge interactions to exert regulatory effects.^[38]^ A recent study found that serum HPA activity was elevated in patients with new coronavirus pneumonia.^[39]^ In an animal experiment, it was shown that HPA levels in the kidneys significantly increased 4 hours after CLP in septic mice, leading to a decline in renal function. This suggests that inhibiting HPA could potentially prevent sepsis-induced AKI.^[32]^ Acute gastrointestinal dysfunction caused by sepsis is intricately linked to HPA.^[40]^ In sepsis model mice, a significant degradation of heparan sulfate (HS) was observed in the intestinal villi, while levels of HPA in the intestinal tissue increased, along with elevated levels of inflammatory factors.^[41]^

During sepsis, high expression of HPA leads to the release of numerous inflammatory factors, which in turn enhances the interaction between immune and endothelial cells, resulting in a circulating inflammatory response.^[42]^ Simultaneously, HPA-induced damage to eGC can increase vascular permeability, leading to interstitial edema, and promote the coagulation and adhesion of cytokines, ultimately causing microcirculation disturbance.^[43]^ HPA degrades a notable number of circulating HS fragments, leading to a decrease in the expression of mitochondrial receptors during sepsis. This, in turn, results in mitochondrial damage and cardiomyocyte apoptosis.^[44]^ HPA also enhances the expression of transforming growth factor β (TGF-β), fibroblast growth factor-2 (FGF-2) and matrix metalloproteinases (MMPs), leading to ECM remodeling and worsening of fibrosis.^[45,46]^ Therefore, it can be inferred that HPA is associated with the pathogenesis of SCM, leading to the development of SCM.

The present study conducted a search on electronic databases such as PubMed and Web of Science, using keywords such as “heparanase, mitochondrial dysfunction, microcirculatory disturbance, fibrosis, septic cardiomyopathy” to understand the involvement of HPA in the pathogenesis of SCM, and its role in inducing its occurrence and progression.

## 2. Heparanase (HPA)

HPA-1 is a specific glucuronidase that can participate in cell proliferation, invasion and signal transduction.^[44]^ The concentration of HPA-1 was found to be significantly increased in the blood of patients with sepsis.^[47]^ It is also a widely studied type and can be called HPA.^[47]^ Therefore, this article focuses on describing HPA. HPA is classified as a carbohydrate-processing enzyme and is specifically categorized as a member of the glycoside hydrolase 79 family.^[47]^ Its main function is to catalyze the hydrolysis of internal glucuronic acid β1–4 N-sulfoglucosamine linkages in HS.^[47]^ The HPA gene consists of 14 exons and 13 introns, and is located on chromosome 4q21.3. Through alternative splicing, HPA produces 2 different mRNA transcripts, both of which contain the same open reading frame.^[48]^ Under physiological conditions, HPA can be expressed in platelets, placenta and immune cells.^[49]^ When sepsis occurs, endothelial cells can produce a large quantity of HPA, which can be cleaved from the 65-kDa proenzyme to the 50-kDa form under the action of inflammatory factors and cathepsin L. Activation of HPA can lead to the shedding of eGC, causing endothelial damage and capillary leakage, promoting the occurrence of inflammation and the transfer of inflammatory cells, finally leading to severe complications of sepsis.^[50]^ HPA-2 is a molecule with the same sequence as HPA-1, but its physiological role has not yet been clearly elucidated.^[48]^ It has been shown that HPA-2 is produced by endothelial cells, and its levels in the body are related to syndecan-1.^[51]^ HPA-2 has a higher affinity for HS and heparin, and, unlike HPA-1, HPA-2 has no enzymatic activity.^[37]^ HPA-2 can inhibit the activity of HPA-1, reduce the production of IL-6 in endothelial cells, and exert anti-inflammatory effects, but the specific mechanism of HPA-2 in sepsis requires further investigation.^[51]^

HPA is a multifunctional protein that exhibits both enzymatic and non-enzymatic activities. Its primary physiological role involves the degradation of HSPG on the cell surface,^[52]^ which subsequently affects the structure of the basement membrane and ECM. Additionally, active HPA can also cause a large quantity of cytokines, growth factors and lipoproteins to be released through the ECM, as well as promote cell migration, accelerate angiogenesis, participate in inflammation and coagulation reactions, and cause autophagy and exosome production.^[52]^ In addition, the C-domain in HPA exhibits non-enzymatic activity,^[53]^ including enhancing intercellular adhesion^[54]^ and inducing the phosphorylation of p38 and SRC,^[55]^ which are associated with the vascular endothelial growth factor (VEGF)^[48]^ and tissue factor (TF) genes.^[44]^ Non-enzymatically active HPA is also involved in signaling pathways that activate protein kinases A and C, enabling lysosomes to secrete the active form of HPA.^[56]^ Non-enzymatic HPA can induce endothelial cell invasion and migration through the PI3K/Akt signaling pathway.^[57]^ Therefore, the enzymatic activity of HPA is crucial in inflammation, coagulation, autophagy and exosome formation. On the other hand, the non-enzymatic activity of HPA also plays an important role in promoting angiogenesis and intercellular adhesion. The occurrence of sepsis is associated with inflammation, coagulation and other reactions. A large quantity of HPA is released in the inflammatory state, which further promotes the inflammatory response, activates coagulation factors and promotes clotting activity. Therefore, HPA is closely related to the occurrence and development of sepsis.

## 3. Possible pathophysiological changes caused by HPA in SCM

**Figure 2.**
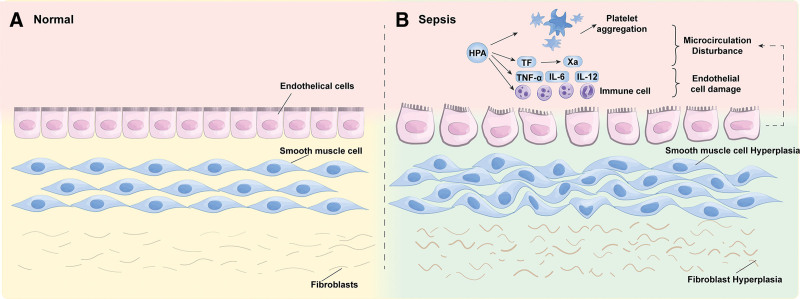
Possible pathophysiological changes caused by heparanase in septic cardiomyopathy. HPA may induce SCM through various pathophysiological changes including mediating inflammation and immune response, microcirculation disturbance, mitochondrial dysfunction, and fibrosis. HPA = heparanase, SCM = septic cardiomyopathy.

HPA may induce SCM through various pathophysiological changes including mediating inflammation and immune response, microcirculation disturbance, mitochondrial dysfunction and fibrosis (Fig. 2). The present study aimed to elaborate and summarize the pathophysiological changes associated with HPA’s involvement in SCM focusing on these aspects, with the goal of contributing to a better understanding of the pathogenesis, diagnosis and treatment of SCM (Table 1).

**Table 1:** Possible pathophysiological changes caused by HPA in SCM.

Path	Related Biomaker	HPA Function	Expression in SCM	References
HPA promotes SCM through inflammation and immune response	TNF-α	Promoted	Increased	^[52,58]^
	IL-6, IL-12	Promoted	Increased	^[58]^
	NO	Promoted inhibition	Increased/decreased	^[53–56]^
HPA promotes SCM through microcirculation disturbance	TF	Promoted	Increased	^[58,59]^
	TFPI	Dissociated	Increased/decreased	^[60]^
	Xa	Promoted	Increased	^[59]^
	VEGF	Promoted	Increased/decreased	^[61,62]^
HPA promotes SCM through mitochondrial dysfunction	PGC-1α	Inhibition	Increased	^[63]^
	TLR-4	Inhibition	Decreased	^[51,64,65]^
	LC3II	Promoted	Increased	^[66]^
	mTOR	Inhibition	Increased	^[67–69]^
HPA promotes SCM through fibrosis	TGF-β	Promoted	Increased	^[31,66]^
	FGF-2	Promoted	Increased	^[45]^
	MMPs	Promoted	Increased	^[30,46]^

*HPA promotes SCM through inflammation and immune response.* Activated macrophages play a crucial role in the release of TNF, an important mediator in endotoxin-induced septic shock. TNF is also released by cardiomyocytes.^[52]^ Furthermore, IL-1 is synthesized in response to circulating TNFs, which are released by macrophages, neutrophils and monocytes. IL-1 inhibits myocardial contractility through the stimulation of nitric oxide (NO).^[53]^ In summary, inflammatory factors play an important role in the early reduction of myocardial contractility. NO is generated through the oxidation of L-arginine by NO synthase (NOS), which is expressed in cardiomyocytes.^[54]^ Overexpression of inducible NOS (iNOS) is a key factor in vasodilation and hypotension during shock.^[55]^ In sepsis, excessive activation of iNOS in cardiomyocytes and immune cells leads to the production of circulating NO, which negatively impacts myocardial contractile function.^[56]^

The immune system’s homeostasis plays a crucial role in the development and progression of sepsis.^[57]^ For instance, research has shown that the activation of the complement system and the increase in extracellular histones are also involved in the pathogenesis of SCM, which can further damage cardiomyocytes.^[15]^ Moreover, sepsis leads to the release of numerous inflammatory factors, which stimulate endothelial cells to secrete a large number of adhesion molecules, and enhance the interaction between leukocytes and endothelial cells.^[70]^ Pro-inflammatory cytokines attract activated monocytes and macrophages, which then migrate into the space between endothelial cells, causing injury to the myocardial endothelium.^[71]^

Active HPA is present in activated leukocytes or proliferating endothelial cells. It cleaves HS chains and enhances the shedding of cell surface proteoglycans, including syndecan-1, thus promoting the proliferation and migration of inflammatory cells.^[72,73]^ HS plays a crucial role in the inflammatory response by regulating the interaction between leukocytes and the vascular endothelium. It promotes the recruitment and rolling of leukocytes, thereby controlling the release of inflammatory cytokines.^[74]^ HPA, derived from endothelial cells, has been found to promote the migration of subendothelial lymphocytes, dendritic cells and leukocytes through the ECM.^[75]^ Leukocyte-produced HPA stimulates multiple cells, leading to intercellular adhesion and upregulation of inflammatory factors.^[60,76]^ Moreover, HPA enhances the activation of macrophages in vitro through LPS, resulting in increased production of TNF-α, IL-6, IL-12 and other cytokines. Activated macrophages, in turn, induce epithelial HPA expression and sustain the inflammatory cycle by increasing the secretion of cathepsins.^[58]^ Conversely, inhibiting HPA activity can reduce inflammatory cell infiltration, protect cell mucosal integrity and suppress the inflammatory response.^[59]^

In conclusion, HPA may induce the occurrence of SCM by cleaving the HS chain, accelerating the shedding of syndecan-1, promoting the migration of inflammatory cells, and regulating the interaction between immune cells and endothelial cells, leading to an inflammatory cascade reaction. However, further research is required to fully understand the specific mechanisms.

*HPA promotes SCM through microcirculation disturbance.* Abnormal coronary endothelial function has been identified as the primary factor contributing to changes in coronary microcirculation blood flow, ultimately leading to myocardial dysfunction.^[61]^ The eGC, which is present on the luminal surface of endothelial cells in all vascular beds, not only regulates endothelial function,^[62]^ but also plays a crucial role in signal transduction.^[77]^ Specifically, it plays a key role in maintaining coronary microcirculation homeostasis.^[63,78]^ It has been reported that coronary microvascular endothelial cells’ glycocalyx damage in a type 1 diabetes model causes increased coronary microcirculation permeability, myocardial edema and ventricular diastolic dysfunction.^[79]^ In addition, disturbances in microvascular blood flow can also trigger eGC shedding to impair myocardial oxygen delivery. Disruption of eGCs may also affect NO signaling, promoting interactions between platelets, leukocytes and endothelial cells.^[79]^ After restoration of eGC and microvascular permeability, reversible changes in ventricular diastolic dysfunction and myocardial edema were also improved in a short period of time.^[70]^ For example, MMPs and inhibitors can attenuate eGC damage, and other endothelial growth factors such as VEGF can also restore eGC, although they have certain limitations.^[71–73]^

HPA-mediated loss of eGCs leads to vascular hyperpermeability, interstitial edema and enhanced neutrophil adhesion to vascular surfaces during sepsis.^[74,75]^ Desquamated eGCs lead to dysfunction of the secondary vascular barrier by creating open cracks in the endothelial layer.^[60,76]^ When endothelial cells lack eGC, they can attract platelets to their inner surface, trigger intravascular coagulation and hinder the anticoagulant function of the endothelium.^[76]^ It has been reported that HPA may act as a cofactor of TF to directly activate related clotting factors, and further enhance the expression and activity of TF.^[58]^ HPA can also directly activate TF and generate factor Xa, enhance the dissociation of TF pathway inhibitor from the cell surface, and form disseminated intravascular coagulation (DIC).^[59]^ In addition, HPA may promote angiogenesis by inducing the expression of angiogenesis-promoting factors such as VEGF and TF to reduce angiogenesis-inhibiting factors.^[61,62]^ It has been reported that the natural anticoagulant heparin exerts anticoagulant activity by competing with HS to bind to HPA to prevent platelet coagulation around cells.^[77]^

Therefore, coronary endothelial cell injury and eGC shedding may cause microcirculation disturbance. HPA can also lead to coagulation system disorders, resulting in DIC. Therefore, HPA may cause SCM through microcirculation disturbance, although the specific mechanism is not clear and needs to be further explored.

*HPA promotes SCM through mitochondrial dysfunction.* Mitochondrial biogenesis is the growth, division and production of mitochondria to meet the metabolic needs of the cell.^[78]^ Mitochondrial biogenesis can be affected by the activation of peroxisome proliferator-activated receptor-coactivators (PGC), primarily PGC-1α and PGC-1β.^[63]^ A previous study showed that the inhibition of PGC-1α in late-stage SCM and LPS-treated cardiomyocytes caused metabolic disturbances in cardiomyocytes, resulting in decreased ventricular function, while activation of PGC-1α reduced cardiomyocyte apoptosis and protected cardiomyocytes from damage.^[79]^

Mitophagy is important for maintaining mitochondrial biogenesis and a crucial regulatory mechanism for cells to specifically remove damaged mitochondria. Two pathways may be involved in the identification and elimination of dysfunctional mitochondria: One is the substrate-specific PINK1/Parkin pathway that mediates mitochondrial ubiquitination. It can be recognized by the adapter protein p62 and binds to LC3; the second pathway is a ubiquitin-independent pathway involving direct binding of ATG8 family proteins to autophagy receptors.^[80]^ A previous study found that overexpression of the cardiac proteins ATG5, ATG7, LC3II, and p62 during sepsis increased autophagy levels,^[81]^ and numerous damaged mitochondria generated great quantities of ROS and induced cardiomyocyte apoptosis. Therefore, mitophagy at physiological levels maintains mitochondrial homeostasis, while excessive mitophagy may reduce ATP and induce apoptosis.^[80]^

HS fragments produced by HPA degradation have been reported to reduce the expression of mitochondrial functional receptors, such as PGC-1α.^[64]^ Since HS regulates the TLR-4 signaling pathway and its downstream inflammatory response,^[51]^ inhibition of TLR-4 can antagonize the low expression of PGC-1α, demonstrating that HS is involved in mitochondrial dysfunction.^[64,65]^ Autophagy promotes survival under physiological conditions, whereas, under pathological conditions, it may promote cell death.^[37]^ HPA localizes directly in autophagosomes and stimulates autophagy formation by a non-enzymatic mechanism.^[67]^ It has been reported that LC3II levels are increased in transgenic mice overexpressing HPA, whereas LC3II levels can be reduced in HPA-knockout mice.^[66]^ However, the mechanism of HPA-induced autophagy is not yet fully understood, and it may be related to mTOR.^[68]^ HPA downregulates mTOR signaling to induce autophagy, and mTOR activity can be quantified by reduced p70 S6 kinase phosphorylation levels.^[67,69]^

Therefore, HPA may cause mitochondrial damage by degrading HS fragments and reducing the expression of mitochondrial functional receptors, leading to the generation of SCM. In addition, under pathological conditions, HPA may excessively induce autophagy, leading to damage of numerous mitochondria, causing myocardial damage and further leading to SCM.

*HPA promotes SCM through fibrosis.* Fibroblasts and myofibroblasts are responsible for ECM protein synthesis, and act as key effectors of fibrosis in multiple organs.^[82]^ Cardiac fibroblasts can aggravate cardiomyocyte aging through paracrine function and ECM remodeling.^[83]^ During sepsis, cardiomyocytes secrete a variety of paracrine factors, including ILS, TGF-β, β-2 microglobulin, FGF, placental growth factor, and mediate the communication between fibroblasts and cardiomyocytes.^[31]^ Among them, TGF-β is involved in multiple aspects of fibrosis, including activation of myofibroblasts and remodeling of the ECM. Increased expression of TGF-β has been found in cardiomyocytes of both dilated and hypertrophic cardiomyopathy.^[66]^ MMPs mutate and degrade fibrillar collagen and other components of the ECM, and dysregulation of MMP activity remodels the myocardial ECM and promotes myocardial fibrosis.^[30]^ It has been reported that mice lacking MMP-9 and MMP-2 can prevent myocardial damage, relieve left ventricular dilatation and collagen accumulation, and reduce myocardial fibrosis after acute myocardial infarction.^[84]^ In summary, cardiac fibrosis, which is characterized by an accumulation of ECM proteins in the myocardial interstitium, causing myocardial wall thickening, and systolic or diastolic dysfunction, and impairing overall cardiac function, is an inevitable consequence of chronic myocardial injury.^[85]^

HPA can increase the expression of TGF-β and FGF-2, which activate PI3K/AKT to continue to upregulate their own expression and accelerate the remodeling of ECM.^[45]^ MMPs are a family of zinc-containing enzymes, and disorder of MMPs is also involved in myocardial ECM remodeling and development of myocardial fibrosis.^[30]^ Previous studies have shown that HPA upregulates the expression of MMPs through the p38 MAPK signaling pathway and aggravates fibrosis through the TGF-β signaling pathway.^[30,46]^ High-mobility histone B1 can activate fibroblasts through its receptor RAGE-B, and the activated NF continues to upregulate HPA, release TGF stored in ECM by decomposing HS-β and accelerate the process of fibrosis.^[86]^

To summarize, fibrosis is closely related to the occurrence and development of SCM. HPA may accelerate ECM remodeling and promote fibrosis by upregulating fibrosis-related factors, including TGF-β, FGF-2 and MMP, thereby inducing the occurrence of SCM.

## 4. Other possible mechanisms of HPA-induced SCM

**Figure 3.**
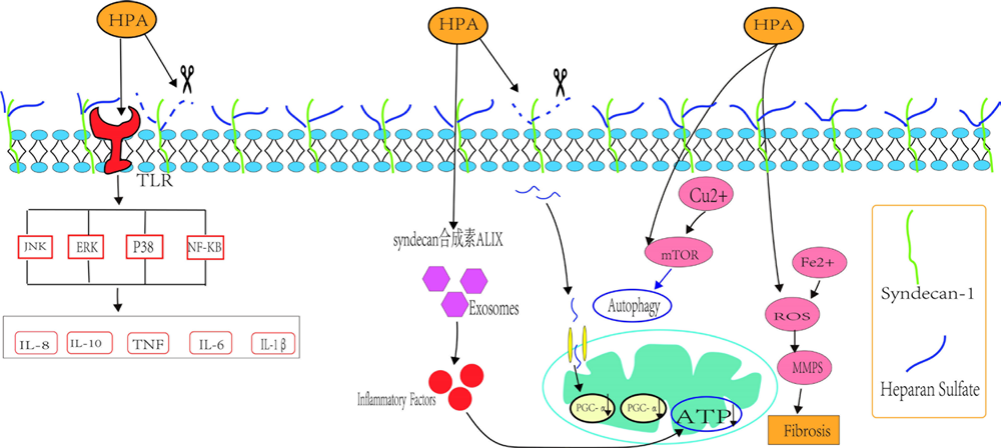
Mechanisms of action of heparanase (HPA). HPA may mediate TLR and cause multi-signal transduction through the MAPK pathway to release inflammatory factors to cause SCM; HPA may activate the syndecan synthin ALIX pathway, promote the production of exosomes, and secrete a variety of inflammatory factors leading to SCM; HPA may cause mitochondrial damage through excessive induction of autophagy, reduce ATP, and cause copper death; HPA may cause ferroptosis through the massive generation of ROS. HPA = heparanase, MAPK = mitogen-activated protein kinase, ROS = reactive oxygen species, SCM = septic cardiomyopathy.

HPA activates TLR-2/TLR-4 receptors, and stimulates p38, JNK, and ERK signaling through the MAPK pathway, leading to the production of inflammatory factors such as TNF-α, IL-1 and IL-6.^[87]^ HPA may promote the inflammatory response through the above pathways, leading to the occurrence of SCM. Extracellular vesicles are composed of plasma membrane-derived particles of different sizes, which are secreted outside the cell, and the exocytosis of multivesicular bodies produces vesicles called exosomes.^[88]^ HPA has been shown to localize on the surface of exosomes,^[89]^ and it activates the syndecan synthin ALIX pathway to promote exosome production.^[90]^ Macrophage-derived exosomes were able to recognize long-chain fatty acids, activate the downstream ERK signaling pathway, promote the secretion of inflammatory factors (such as IL-1β and TNF-α), trigger a systemic inflammatory cascade response, and lead to a decrease in mitochondrial ATP production and calcium sensitivity of cardiomyocytes, eventually causing myocardial dysfunction.^[91]^ A decreased number of copper ions in cardiomyocytes can lead to reduced cardiac contractility and energy failure, while an elevated number of copper ions can mediate cardiomyocyte death and cardiac fibrosis, and have a positive correlation with ROS generation.^[92]^ CuSO4 induces mitophagy through the mTOR pathway, increases excessive generation of mitochondrial ROS and leads to SCM.^[93]^ HPA also induces autophagy by inhibiting mTOR.^[90]^ Therefore, under pathological conditions, HPA may cause mitochondrial damage, ATP reduction, copper death induction, and SCM promotion by excessive induction of autophagy. Ferroptosis is a unique form of death based on ROS. When the Fe2 + level in heart cells increases, the body can produce a large quantity of ROS, which can damage cardiomyocytes through the Fenton reaction, aggravate ferroptosis and cause myocardial damage.^[94]^ Excessive ROS production by HPA in an inflammatory state causes mitochondrial damage and cardiomyocyte death.^[95]^ Therefore, HPA may induce ferroptosis through large production of ROS and promote SCM. In summary, HPA may mediate TLR and cause multi-signal transduction through the MAPK signaling pathway to release inflammatory factors to cause SCM. HPA may also activate the syndecan synthin ALIX pathway, promote the production of exosomes, and secrete a variety of inflammatory factors leading to SCM. Furthermore, HPA may cause mitochondrial damage through excessive induction of autophagy, reduce ATP and cause copper death. HPA may cause ferroptosis through increased generation of ROS. The possible pathogenic mechanism of HPA in promoting SCM needs further exploration (Fig. 3).

## 5. Summary

HPA plays an important role in the pathogenesis of SCM, and may induce SCM through inflammation and immunity, and by participating in microcirculation disturbance, mitochondrial dysfunction and promoting fibrosis. In addition, HPA may also indirectly affect ferroptosis and copper death pathways to promote SCM production by promoting exosome production. In the future, further investigation into the pathogenesis, diagnosis and treatment of SCM may lead to new targets. HPA inhibitors could potentially be a promising avenue to reduce SCM mortality. However, it is important to note that there is limited research on the association between HPA and SCM, thus emphasizing the need for continued investigation in this area.

## Author contributions

**Investigation:** Lin-Jun Wang.

**Supervision:** Hong-Lei Li, Jian-Chun Li, Liping Liu.

**Writing – original draft:** Di Chen.

**Writing – review & editing:** Fei Feng, Liping Liu.

## References

[1] Cimolai MC Alvarez S Bode C Bugger H. Mitochondrial mechanisms in septic cardiomyopathy. Int J Mol Sci. 2015;16:17763–78. 26247933 10.3390/ijms160817763 PMC4581220

[2] Lu NF Jiang L Zhu B. Elevated plasma histone H4 levels are an important risk factor in the development of septic cardiomyopathy. Balkan Med J. 2020;37:72–8. 31674172 10.4274/balkanmedj.galenos.2019.2019.8.40 PMC7094183

[3] Ehrman RR Sullivan AN Favot MJ. Pathophysiology, echocardiographic evaluation, biomarker findings, and prognostic implications of septic cardiomyopathy: a review of the literature. Crit Care. 2018;22:112. 29724231 10.1186/s13054-018-2043-8 PMC5934857

[4] Shen XD Zhang H-S Zhang R. Progress in the clinical assessment and treatment of myocardial depression in critically ill patient with sepsis. J Inflamm Res. 2022;15:5483–90. 36164659 10.2147/JIR.S379905 PMC9508933

[5] Beesley SJ Weber G Sarge T. Septic cardiomyopathy. Crit Care Med. 2018;46:625–34. 29227368 10.1097/CCM.0000000000002851

[6] Lin H Wang W Lee M Meng Q Ren H. Current status of septic cardiomyopathy: basic science and clinical progress. Front Pharmacol. 2020;11:210. 32194424 10.3389/fphar.2020.00210 PMC7062914

[7] Martin L Derwall M Al Zoubi S. The septic heart: current understanding of molecular mechanisms and clinical implications. Chest. 2019;155:427–37. 30171861 10.1016/j.chest.2018.08.1037

[8] de Braga Lima Carvalho Canesso M Borges IN de Deus Queiroz Santos TA. Value of speckle-tracking echocardiography changes in monitoring myocardial dysfunction during treatment of sepsis: potential prognostic implications. Int J Cardiovasc Imaging. 2019;35:855–9. 30847658 10.1007/s10554-018-01525-1

[9] Jeong HS Lee TH Bang CH Kim J-H Hong SJ. Risk factors and outcomes of sepsis-induced myocardial dysfunction and stress-induced cardiomyopathy in sepsis or septic shock: a comparative retrospective study. Medicine (Baltim). 2018;97:e0263. 10.1097/MD.0000000000010263 PMC5895365 29595686

[10] Wang J Gao X He Z Wang J Xu G Li T. Evaluating the effects of Esmolol on cardiac function in patients with Septic cardiomyopathy by Speck-tracking echocardiography-a randomized controlled trial. BMC Anesthesiol. 2023;23:51. 36765286 10.1186/s12871-023-01983-8 PMC9912519

[11] Wang X Su L Yang R Zhang H Liu D. Myocardial strain/stress changes identified by echocardiography may reveal early sepsis-induced myocardial dysfunction. J Int Med Res. 2018;46:1439–54. 29332410 10.1177/0300060517737434 PMC6091847

[12] Lang RM Badano LP Mor-Avi V. Recommendations for cardiac chamber quantification by echocardiography in adults: an update from the American Society of Echocardiography and the European Association of, Cardiovascular Imaging. Eur Heart J Cardiovasc Imaging. 2016;17:412. 26983884 10.1093/ehjci/jew041

[13] Bansal M Mehta A Machanahalli Balakrishna A. Right ventricular dysfunction in sepsis: an updated narrative review. Shock. 2023;59:829–37. 36943772 10.1097/SHK.0000000000002120

[14] Vallabhajosyula S Shankar A Vojjini R. Impact of right ventricular dysfunction on short-term and long-term mortality in sepsis: a meta-analysis of 1,373 patients. Chest. 2021;159:2254–63. 33359215 10.1016/j.chest.2020.12.016 PMC8579312

[15] Li J Sun G Ma H. Identification of immune-related hub genes and miRNA-mRNA pairs involved in immune infiltration in human septic cardiomyopathy by bioinformatics analysis. Front Cardiovasc Med. 2022;9:971543. 36204577 10.3389/fcvm.2022.971543 PMC9530044

[16] Sato R Nasu M. A review of sepsis-induced cardiomyopathy. J Intensive Care. 2015;3:48. 26566443 10.1186/s40560-015-0112-5 PMC4642671

[17] Zhang ZC Dai HW Yu YH Yang JD Hu CB. Usefulness of heart-type fatty acid-binding protein in patients with severe sepsis. J Crit Care. 2012;27:415.e13–8. 10.1016/j.jcrc.2012.01.004 22386224

[18] Zhang Z Dai H Yu Y Yang J Chen J Wu L. Elevated pregnancy-associated plasma protein A predicts myocardial dysfunction and death in severe sepsis. Ann Clin Biochem. 2014;51(Pt 1):22–9. 23880622 10.1177/0004563213489275

[19] Zeng N Xu J Yao W Li S Ruan W Xiao F. Brain-derived neurotrophic factor attenuates septic myocardial dysfunction via eNOS/NO pathway in rats. Oxid Med Cell Longev. 2017;2017:1721434. 28770018 10.1155/2017/1721434 PMC5523440

[20] Rosengart MR Nathens AB Arbabi S. Mitogen-activated protein kinases in the intensive care unit: prognostic potential. Ann Surg. 2003;237:94–100. 12496535 10.1097/00000658-200301000-00013 PMC1513967

[21] Kalbitz M Fattahi F Grailer JJ. Complement-induced activation of the cardiac NLRP3 inflammasome in sepsis. FASEB J. 2016;30:3997–4006. 27543123 10.1096/fj.201600728R PMC5102118

[22] Ince C Mayeux PR Nguyen T. ADQI XIV Workgroup. The endothelium in sepsis. Shock. 2016;45:259–70. 26871664 10.1097/SHK.0000000000000473 PMC5281063

[23] Benador IY Veliova M Liesa M Shirihai OS. Mitochondria bound to lipid droplets: where mitochondrial dynamics regulate lipid storage and utilization. Cell Metab. 2019;29:827–35. 30905670 10.1016/j.cmet.2019.02.011 PMC6476311

[24] Nakamura H Matoba S Iwai-Kanai E. p53 promotes cardiac dysfunction in diabetic mellitus caused by excessive mitochondrial respiration-mediated reactive oxygen species generation and lipid accumulation. Circ Heart Fail. 2012;5:106–15. 22075967 10.1161/CIRCHEARTFAILURE.111.961565

[25] Tsushima K Bugger H Wende AR. Mitochondrial reactive oxygen species in lipotoxic hearts induce post-translational modifications of AKAP121, DRP1, and OPA1 that promote mitochondrial fission. Circ Res. 2018;122:58–73. 29092894 10.1161/CIRCRESAHA.117.311307 PMC5756120

[26] Ducasa GM Mitrofanova A Fornoni A. Crosstalk between lipids and mitochondria in diabetic kidney disease. Curr Diab Rep. 2019;19:144. 31754839 10.1007/s11892-019-1263-x PMC12862944

[27] Liu YC Yu M-M Shou S-T Chai Y-F. Sepsis-induced cardiomyopathy: mechanisms and treatments. Front Immunol. 2017;8:1021. 28970829 10.3389/fimmu.2017.01021 PMC5609588

[28] Hollenberg SM Singer M. Pathophysiology of sepsis-induced cardiomyopathy. Nat Rev Cardiol. 2021;18:424–34. 33473203 10.1038/s41569-020-00492-2

[29] Weiss SL Zhang D Bush J. Persistent mitochondrial dysfunction linked to prolonged organ dysfunction in pediatric sepsis. Crit Care Med. 2019;47:1433–41. 31385882 10.1097/CCM.0000000000003931 PMC7341116

[30] Chen LC Shibu MA Liu C-J. ERK1/2 mediates the lipopolysaccharide-induced upregulation of FGF-2, uPA, MMP-2, MMP-9 and cellular migration in cardiac fibroblasts. Chem Biol Interact. 2019;306:62–9. 30980805 10.1016/j.cbi.2019.04.010

[31] Tang X Li PH Chen HZ. Cardiomyocyte senescence and cellular communications within myocardial microenvironments. Front Endocrinol (Lausanne). 2020;11:280. 32508749 10.3389/fendo.2020.00280 PMC7253644

[32] Zarbock A Nadim MK Pickkers P. Sepsis-associated acute kidney injury: consensus report of the 28th Acute Disease Quality Initiative workgroup. Nat Rev Nephrol. 2023;19:401–17. 36823168 10.1038/s41581-023-00683-3

[33] Liu D Huang S-Y Sun J-H. Sepsis-induced immunosuppression: mechanisms, diagnosis and current treatment options. Mil Med Res. 2022;9:56. 36209190 10.1186/s40779-022-00422-y PMC9547753

[34] Stasi A Fiorentino M Franzin R. Beneficial effects of recombinant CER-001 high-density lipoprotein infusion in sepsis: results from a bench to bedside translational research project. BMC Med. 2023;21:392. 37915050 10.1186/s12916-023-03057-5 PMC10621167

[35] Sinkovic A Kit B Markota A. Successful use of combined blood purification techniques in splenectomised patient with septic shock in streptococcus pneumoniae infection - a case report. BMC Infect Dis. 2018;18:433. 30157806 10.1186/s12879-018-3327-y PMC6114280

[36] De Rosa S Marengo M Fiorentino M. SIAARTI-SIN joint commission. Extracorporeal blood purification therapies for sepsis-associated acute kidney injury in critically ill patients: expert opinion from the SIAARTI-SIN joint commission. J Nephrol. 2023;36:1731–42. 37439963 10.1007/s40620-023-01637-5 PMC10543830

[37] Vlodavsky I Gross-Cohen M Weissmann M Ilan N Sanderson RD. Opposing functions of heparanase-1 and heparanase-2 in cancer progression. Trends Biochem Sci. 2018;43:18–31. 29162390 10.1016/j.tibs.2017.10.007 PMC5741533

[38] Yuan F Yang Y Zhou H. Heparanase in cancer progression: structure, substrate recognition and therapeutic potential. Front Chem. 2022;10:926353. 36157032 10.3389/fchem.2022.926353 PMC9500389

[39] Mingoti MED Bertollo AG Simões JLB Francisco GR Bagatini MD Ignácio ZM. COVID-19, oxidative stress, and neuroinflammation in the depression route. J Mol Neurosci. 2022;72:1166–81. 35322375 10.1007/s12031-022-02004-y PMC8942178

[40] Chen TT Lv J-J Chen L Li M Liu L-P. Heparanase inhibition leads to improvement in patients with acute gastrointestinal injuries induced by sepsis. World J Gastroenterol. 2023;29:5154–65. 37744293 10.3748/wjg.v29.i35.5154 PMC10514756

[41] Chen TT Lv J-J Chen L Gao Y-W Liu L-P. Role of heparinase in the gastrointestinal dysfunction of sepsis (Review). Exp Ther Med. 2022;23:119. 34970342 10.3892/etm.2021.11042 PMC8713170

[42] Vlodavsky I Beckhove P Lerner I. Significance of heparanase in cancer and inflammation. Cancer Microenviron. 2012;5:115–32. 21811836 10.1007/s12307-011-0082-7 PMC3399068

[43] Putz EM Mayfosh AJ Kos K. NK cell heparanase controls tumor invasion and immune surveillance. J Clin Invest. 2017;127:2777–88. 28581441 10.1172/JCI92958 PMC5490772

[44] Pape T Hunkemöller AM Kümpers P Haller H David S Stahl K. Targeting the “sweet spot” in septic shock - A perspective on the endothelial glycocalyx regulating proteins Heparanase-1 and -2. Matrix Biol Plus. 2021;12:100095. 34917926 10.1016/j.mbplus.2021.100095 PMC8669377

[45] Masola V Zaza G Onisto M Lupo A Gambaro G. Impact of heparanase on renal fibrosis. J Transl Med. 2015;13:181. 26040666 10.1186/s12967-015-0538-5 PMC4467599

[46] Liu X Zhou Z-H Li W. Heparanase promotes tumor growth and liver metastasis of colorectal cancer cells by activating the p38/MMP1 axis. Front Oncol. 2019;9:216. 31001480 10.3389/fonc.2019.00216 PMC6454005

[47] Wu L Viola CM Brzozowski AM Davies GJ. Structural characterization of human heparanase reveals insights into substrate recognition. Nat Struct Mol Biol. 2015;22:1016–22. 26575439 10.1038/nsmb.3136 PMC5008439

[48] Levy-Adam F Feld S Cohen-Kaplan V. Heparanase 2 interacts with heparan sulfate with high affinity and inhibits heparanase activity. J Biol Chem. 2010;285:28010–9. 20576607 10.1074/jbc.M110.116384 PMC2934666

[49] Liederman Z Van Cott EM Smock K Meijer P Selby R. Heparin-induced thrombocytopenia: an international assessment of the quality of laboratory testing. J Thromb Haemost. 2019;17:2123–30. 31420903 10.1111/jth.14611

[50] Martin L Koczera P Zechendorf E Schuerholz T. The endothelial glycocalyx: new diagnostic and therapeutic approaches in sepsis. Biomed Res Int. 2016;2016:3758278. 27699168 10.1155/2016/3758278 PMC5028820

[51] Kiyan Y Tkachuk S Kurselis K. Heparanase-2 protects from LPS-mediated endothelial injury by inhibiting TLR4 signalling. Sci Rep. 2019;9:13591. 31537875 10.1038/s41598-019-50068-5 PMC6753096

[52] Masola V Bellin G Gambaro G Onisto M. Heparanase: a multitasking protein involved in Extracellular Matrix (ECM) remodeling and intracellular events. Cells. 2018;7:236. 30487472 10.3390/cells7120236 PMC6316874

[53] Fux L Feibish N Cohen-Kaplan V. Structure-function approach identifies a COOH-terminal domain that mediates heparanase signaling. Cancer Res. 2009;69:1758–67. 19244131 10.1158/0008-5472.CAN-08-1837 PMC2650747

[54] Goldshmidt O Zcharia E Cohen M. Heparanase mediates cell adhesion independent of its enzymatic activity. FASEB J. 2003;17:1015–25. 12773484 10.1096/fj.02-0773com

[55] Zetser A Bashenko Y Miao H-Q Vlodavsky I Ilan N. Heparanase affects adhesive and tumorigenic potential of human glioma cells. Cancer Res. 2003;63:7733–41. 14633698

[56] Shafat I Vlodavsky I Ilan N. Characterization of mechanisms involved in secretion of active heparanase. J Biol Chem. 2006;281:23804–11. 16790442 10.1074/jbc.M602762200

[57] Gingis-Velitski S Zetser A Kaplan V. Heparanase uptake is mediated by cell membrane heparan sulfate proteoglycans. J Biol Chem. 2004;279:44084–92. 15292202 10.1074/jbc.M402131200

[58] Nadir Y Brenner B Fux L Shafat I Attias J Vlodavsky I. Heparanase enhances the generation of activated factor X in the presence of tissue factor and activated factor VII. Haematologica. 2010;95:1927–34. 20634491 10.3324/haematol.2010.023713 PMC2966916

[59] Nadir Y Brenner B Zetser A. Heparanase induces tissue factor expression in vascular endothelial and cancer cells. J Thromb Haemost. 2006;4:2443–51. 16970801 10.1111/j.1538-7836.2006.02212.x

[60] Alphonsus CS Rodseth RN. The endothelial glycocalyx: a review of the vascular barrier. Anaesthesia. 2014;69:777–84. 24773303 10.1111/anae.12661

[61] Abe K Shoji M Chen J. Regulation of vascular endothelial growth factor production and angiogenesis by the cytoplasmic tail of tissue factor. Proc Natl Acad Sci USA. 1999;96:8663–8. 10411932 10.1073/pnas.96.15.8663 PMC17573

[62] Zhang YM Bachmann S Hemmer C. Vascular origin of kaposis-sarcoma - expression of leukocyte adhesion molecule-1 thrombomodulin, and tissue factor. Am J Pathol. 1994;144:51–9. 7507301 PMC1887124

[63] Zhang T Liu C-F Zhang T-N Wen R Song W-L. Overexpression of peroxisome proliferator-activated receptor γ coactivator 1-α protects cardiomyocytes from lipopolysaccharide-induced mitochondrial damage and apoptosis. Inflammation. 2020;43:1806–20. 32529514 10.1007/s10753-020-01255-4

[64] Martin L Peters C Schmitz S. Soluble heparan sulfate in serum of septic shock patients induces mitochondrial dysfunction in murine cardiomyocytes. Shock. 2015;44:569–77. 26529654 10.1097/SHK.0000000000000462

[65] Martin L Schmitz S De Santis R. Peptide 19-2.5 inhibits heparan sulfate-triggered inflammation in murine cardiomyocytes stimulated with human sepsis serum. PLoS One. 2015;10:e0127584. 26024383 10.1371/journal.pone.0127584 PMC4449035

[66] Shteingauz A Boyango I Naroditsky I. Heparanase enhances tumor growth and chemoresistance by promoting autophagy. Cancer Res. 2015;75:3946–57. 26249176 10.1158/0008-5472.CAN-15-0037 PMC4573896

[67] Rabelink TJ van den Berg BM Garsen M Wang G Elkin M van der Vlag J. Heparanase: roles in cell survival, extracellular matrix remodelling and the development of kidney disease. Nat Rev Nephrol. 2017;13:201–12. 28163306 10.1038/nrneph.2017.6

[68] Dunlop EA Tee AR. mTOR and autophagy: a dynamic relationship governed by nutrients and energy. Semin Cell Dev Biol. 2014;36:121–9. 25158238 10.1016/j.semcdb.2014.08.006

[69] Masola V Zaza G Gambaro G Franchi M Onisto M. Role of heparanase in tumor progression: molecular aspects and therapeutic options. Semin Cancer Biol. 2020;62:86–98. 31348993 10.1016/j.semcancer.2019.07.014

[70] Qiu Y Buffonge S Ramnath R. Endothelial glycocalyx is damaged in diabetic cardiomyopathy: angiopoietin 1 restores glycocalyx and improves diastolic function in mice. Diabetologia. 2022;65:879–94. 35211778 10.1007/s00125-022-05650-4 PMC8960650

[71] Ramnath RD Butler MJ Newman G. Blocking matrix metalloproteinase-mediated syndecan-4 shedding restores the endothelial glycocalyx and glomerular fi ltration barrier function in early diabetic kidney disease. Kidney Int. 2020;97:951–65. 32037077 10.1016/j.kint.2019.09.035 PMC7184681

[72] Foster RR Armstrong L Baker S. Glycosaminoglycan regulation by VEGFA and VEGFC of the glomerular microvascular endothelial cell glycocalyx *in Vitro*. Am J Pathol. 2013;183:604–16. 23770346 10.1016/j.ajpath.2013.04.019 PMC3730758

[73] Oltean S Qiu Y Ferguson JK. Vascular endothelial growth factor-A_165_b is protective and restores endothelial glycocalyx in diabetic nephropathy. J Am Soc Nephrol. 2015;26:1889–904. 25542969 10.1681/ASN.2014040350 PMC4520162

[74] Uchimido R Schmidt EP Shapiro NI. The glycocalyx: a novel diagnostic and therapeutic target in sepsis. Crit Care. 2019;23:16. 30654825 10.1186/s13054-018-2292-6 PMC6337861

[75] Eustes AS Campbell RA Middleton EA. Heparanase expression and activity are increased in platelets during clinical sepsis. J Thromb Haemost. 2021;19:1319–30. 33587773 10.1111/jth.15266 PMC8218538

[76] Iba T Levy JH. Derangement of the endothelial glycocalyx in sepsis. J Thromb Haemost. 2019;17:283–94. 30582882 10.1111/jth.14371

[77] Levy-Adam F Abboud-Jarrous G Guerrini M Beccati D Vlodavsky I Ilan N. Identification and characterization of heparin/heparan sulfate binding domains of the endoglycosidase heparanase. J Biol Chem. 2005;280:20457–66. 15760902 10.1074/jbc.M414546200

[78] Wang RZ Xu Y Fang Y. Pathogenetic mechanisms of septic cardiomyopathy. J Cell Physiol. 2022;237:49–58. 34278573 10.1002/jcp.30527

[79] Ravikumar N Sayed MA Poonsuph CJ Sehgal R Shirke MM Harky A. Septic cardiomyopathy: from basics to management choices. Curr Probl Cardiol. 2021;46:100767. 33388489 10.1016/j.cpcardiol.2020.100767

[80] Li GY Li J Shao R Zhao J Chen M. FUNDC1: a promising mitophagy regulator at the mitochondria-associated membrane for cardiovascular diseases. Front Cell Dev Biol. 2021;9:788364. 10.3389/fcell.2021.788634 PMC8797154 35096821

[81] Tan Y Zhang Y He J. Dual specificity phosphatase 1 attenuates inflammation-induced cardiomyopathy by improving mitophagy and mitochondrial metabolism. Mol Metab. 2022;64:101567. 35944900 10.1016/j.molmet.2022.101567 PMC9418987

[82] Secchi MF Masola V Zaza G Lupo A Gambaro G Onisto M. Recent data concerning heparanase: focus on fibrosis, inflammation and cancer. Biomol Concepts. 2015;6:415–21. 26552066 10.1515/bmc-2015-0021

[83] Kamo T Akazawa H Komuro I. Cardiac nonmyocytes in the hub of cardiac hypertrophy. Circ Res. 2015;117:89–98. 26089366 10.1161/CIRCRESAHA.117.305349

[84] Ducharme A Frantz S Aikawa M. Targeted deletion of matrix metalloproteinase-9 attenuates left ventricular enlargement and collagen accumulation after experimental myocardial infarction. J Clin Invest. 2000;106:55–62. 10880048 10.1172/JCI8768 PMC517910

[85] Lafuse WP Wozniak DJ Rajaram MVS. Role of cardiac macrophages on cardiac inflammation, fibrosis and tissue repair. Cells. 2021;10:51. 10.3390/cells10010051 PMC7824389 33396359

[86] Ohayon O Mawasi N Pevzner A. Halofuginone upregulates the expression of heparanase in thioacetamide-induced liver fibrosis in rats. Lab Invest. 2008;88:627–33. 18458672 10.1038/labinvest.2008.30

[87] Koganti R Suryawanshi R Shukla D. Heparanase, cell signaling, and viral infections. Cell Mol Life Sci. 2020;77:5059–77. 32462405 10.1007/s00018-020-03559-y PMC7252873

[88] Zhang Y Huang H Liu W. Endothelial progenitor cells-derived exosomal microRNA-21-5p alleviates sepsis-induced acute kidney injury by inhibiting RUNX1 expression. Cell Death Dis. 2021;12:335. 33785732 10.1038/s41419-021-03578-y PMC8009943

[89] Bandari SK Purushothaman A Ramani VC. Chemotherapy induces secretion of exosomes loaded with heparanase that degrades extracellular matrix and impacts tumor and host cell behavior. Matrix Biol. 2018;65:104–18. 28888912 10.1016/j.matbio.2017.09.001 PMC5816689

[90] Sanderson RD Elkin M Rapraeger AC Ilan N Vlodavsky I. Heparanase regulation of cancer, autophagy and inflammation: new mechanisms and targets for therapy. FEBS J. 2017;284:42–55. 27758044 10.1111/febs.13932 PMC5226874

[91] Carbone F Liberale L Preda A Schindler TH Montecucco F. Septic cardiomyopathy: from pathophysiology to the clinical setting. Cells. 2022;11:2833. 36139408 10.3390/cells11182833 PMC9496713

[92] Masad A Hayes L Tabner BJ. Copper-mediated formation of hydrogen peroxide from the amylin peptide: a novel mechanism for degeneration of islet cells in type-2 diabetes mellitus? FEBS Lett. 2007;581:3489–93. 17617411 10.1016/j.febslet.2007.06.061

[93] Cui XN Wang Y Liu H Shi M Wang J Wang Y. The molecular mechanisms of defective copper metabolism in diabetic cardiomyopathy. Oxid Med Cell Longevity. 2022;2022:1–16. 10.1155/2022/5418376 PMC9553361 36238639

[94] Li N Wang W Zhou H. Ferritinophagy-mediated ferroptosis is involved in sepsis-induced cardiac injury. Free Radic Biol Med. 2020;160:303–18. 32846217 10.1016/j.freeradbiomed.2020.08.009

[95] Gammella E Recalcati S Rybinska I Buratti P Cairo G. Iron-induced damage in cardiomyopathy: oxidative-dependent and independent mechanisms. Oxid Med Cell Longevity. 2015;2015:230182. 10.1155/2015/230182 PMC4387903 25878762

